# Transition-Metal-Related Quantum Emitters in Wurtzite AlN and GaN

**DOI:** 10.1021/acsnano.4c07184

**Published:** 2024-10-12

**Authors:** Kamil Czelej, M. Rey Lambert, Mark E. Turiansky, Aleksei Koshevarnikov, Sai Mu, Chris G. Van de Walle

**Affiliations:** † Faculty of Chemical and Process Engineering, Warsaw University of Technology, Ludwika Warynskiego 1, Warsaw 00-645, Poland; ‡ Institute of Theoretical Physics, Faculty of Physics, University of Warsaw, Warsaw 02-093, Poland; § Department of Physics, University of California, Santa Barbara, California 93106-9530, United States; ∥ Materials Department, University of California, Santa Barbara, California 93106-5050, United States; ⊥ Institute of Organic Chemistry, Polish Academy of Sciences, Kasprzaka 44/52, Warsaw 01-224, Poland; # SmartState Center for Experimental Nanoscale Physics, Department of Physics and Astronomy, University of South Carolina, Columbia, South Carolina 29208, United States

**Keywords:** quantum emitters, transition metals, AlN, GaN, quantum information processing

## Abstract

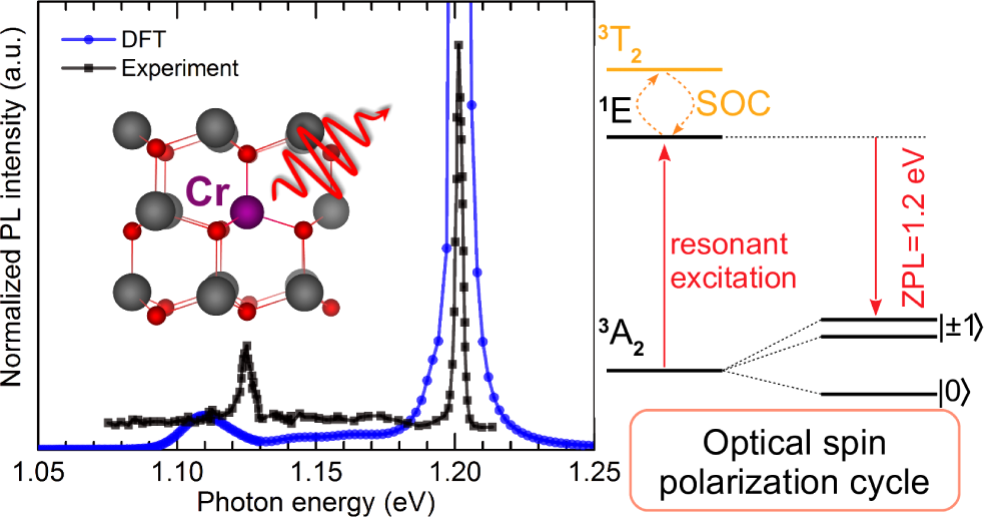

Transition-metal centers exhibit a paramagnetic ground state in wide-bandgap semiconductors and are promising for nanophotonics and quantum information processing. Specifically, there is a growing interest in discovering prominent paramagnetic spin defects that can be manipulated using optical methods. Here, we investigate the electronic structure and magneto-optical properties of Cr and Mn substitutional centers in wurtzite AlN and GaN. We use state-of-the-art hybrid density functional theory calculations to determine level structure, stability, optical signatures, and magnetic properties of these centers. The excitation energies are calculated using the constrained occupation approach and rigorously verified with the complete active space configuration interaction approach. Our simulations of the photoluminescence spectra indicate that  in AlN and  in GaN are responsible for the observed narrow quantum emission near 1.2 eV. We compute the zero-field splitting (ZFS) parameters and outline an optical spin polarization protocol for  and . Our results demonstrate that these centers are promising candidates for spin qubits.

## Introduction

The advancement of quantum technologies is driving investigations of quantum bit (qubit) systems that serve as the fundamental building blocks of quantum information processing.^1,2^ There is strong interest in developing qubits based on quantum defects, i.e., native defects or impurities within the lattice of wide-bandgap materials that provide sufficiently long coherence times of the quantum state.^3^ Various candidates for robust solid-state qubits have been explored, such as the nitrogen vacancy (NV) center in diamond,^4^ the divacancy in SiC,^5^ and various centers in hexagonal BN.^6−9^ The spin initialization and read-out in optically addressable point defects is typically realized by optically detected magnetic resonance (ODMR).^4,10^ The NV center has served as a prototype, but its optical interface suffers from the fact that less than 3% of the emitted photons are in the zero-phonon line (ZPL)—in other words, useful for quantum information—due to strong electron–phonon coupling. Centers based on wave functions that are more isolated from the environment are therefore preferred, which has focused attention on transition-metal impurities in wide-bandgap hosts.^11−13^

3*d* transition-metal impurities in wide-bandgap materials give rise to rich optical, electrical, and magnetic properties that can be exploited in spintronics,^14^ photonics,^15^ and quantum information processing.^11,13,16,17^ Sewani et al.^18^ performed initialization and read-out of the *S* = 3/2 spin state of Cr^3+^ in sapphire (Al_2_O_3_), achieving spin coherence times as high as 3.67 s, and reported an ODMR signal at 36 GHz between the ^4^*A*_2_ ground-state spin levels. Koehl et al. demonstrated ensemble optical spin polarization and ODMR associated with the *S* = 1 electronic ground state of Cr^4+^ in SiC and GaN.^19^ These centers exhibit exceptionally weak electron–phonon coupling, giving rise to very sharp infrared emission,^20^ and enabling optical spin polarization between the ground-state magnetic sublevels and lowest excited state. Transition-metal impurities have also shown large spin-strain couplings that exceed those of common vacancy-related centers, as revealed by acoustically driven magnetic resonance investigations;^21−23^ this renders them promising for mechanical manipulation and control of quantum information. As such, there is a compelling interest to investigate these centers in optoelectronic host materials. AlN and GaN are particularly promising as hosts because of the wide technological base associated with their use in commercial electronic and optoelectronic devices. In addition, the piezoelectric nature of these wurtzite-structure materials makes them a promising platform for mechanical manipulation and control of quantum information.

A transition-metal-based spin qubit needs to satisfy several requirements. Typically, the electron spin is manipulated by applying a pulsed microwave resonance drive. The magnitude of zero-field splitting (ZFS) should therefore be in the range of a few GHz; experiments have demonstrated this can be achieved for some transition-metal centers.^19,24,25^ In addition, emission into the ZPL should be optimized, which requires minimizing the electron–phonon coupling. Finally, spin selective decay from the lowest excited state to the ground state is required.

Here, we investigate the ground- and excited-state properties of chromium and manganese substitutional centers in wurtzite AlN and GaN, i.e., Cr_X_ and Mn_X_ where X = Al or Ga, by applying first-principles methods. We use density functional theory (DFT) with a hybrid functional to calculate the ground-state structure of the defects, including their formation energy and charge-state transition levels. Next we characterize the electronic structure with group theory based on the Kohn–Sham (KS) states; for multideterminant excited states we express the energy as a sum over single-Slater-determinant states calculated with constrained DFT. We compare these results with Tanabe–Sugano diagrams,^26^ and corroborate them with calculations based on the complete active space configuration interaction (CASCI) method for a cluster model. Finally, we analyze the magneto-optical and vibronic properties of the investigated centers and demonstrate the optical spin polarization cycle within the ODMR scheme.

We will show that  in AlN and  in GaN are responsible for the narrow and bright infrared single photon emission observed experimentally around 1.2 eV. In addition, our discussion of the physics of these centers provides useful insights that are more general than the specific defects addressed here. We find that a ground-state configuration that minimizes spin–orbit coupling (SOC) is essential for having ZFS values in the desired range. We also find that the presence of a Jahn–Teller effect leads to strong atomic distortions that reduce emission into the ZPL. Minimizing these SOC and Jahn–Teller effects can be realized with a ground state that is composed of an orbital singlet and spin multiplet, expressed as ^2*S*+1^*A*, where *S* > 0. To enable spin selective decay to the ground state, the lowest excited state should then be an orbital multiplet and low spin state, such as ^1^*E* or ^2^*E*.

Overall, we find that  in AlN and  in GaN satisfy these requirements and are attractive candidates for nanophotonics and quantum information processing applications.

## Results and Discussion

### Formation of Cr_X_ and Mn_X_ Defects and Their Stability in AlN and GaN

**Figure 1:**
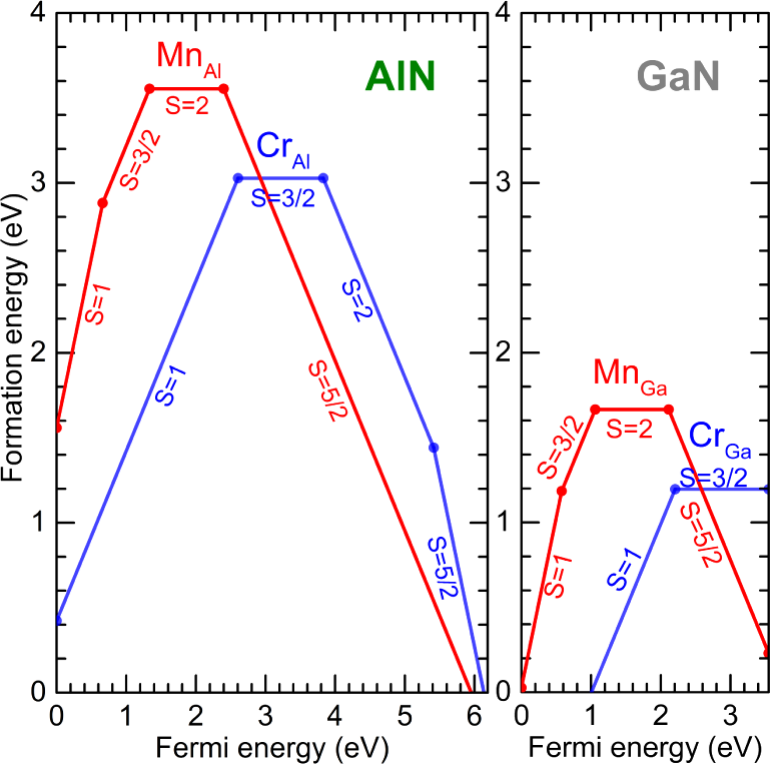
Formation energies as a function of Fermi level for Cr_X_ and Mn_X_ centers in AlN and GaN, assuming cation-rich conditions. The zero of energy is set at the VBM. The slope of the line segments correspond to the charge state. The spin state for each individual charge state is indicated.

The calculated formation energies and charge-state transition levels of Cr_X_ and Mn_X_ centers in AlN and GaN are shown in Figure 1. The formation energy determines the concentration of a defect in equilibrium. If conditions are close to equilibrium (which is the case during high-temperature growth or annealing) only point defects with low enough formation energy will occur in large concentrations. The formation energy diagrams also provide information about the relative stability of a given point defect in various charge states with respect to other defects, and which charge states (and, correspondingly, spin states) a defect acquires at a given Fermi level. Figure 1 demonstrates that the investigated defects are electrically active and can, in principle, occur in positive, neutral, and negative charge states.

Substitutional Cr in GaN, Cr_Ga_, can occur in positive and neutral charge states, with a (+/0) charge-state transition level at 2.21 eV above the VBM. Experimentally, an absorption onset of 1.5 eV was reported for unintentionally Cr-doped GaN, in reasonable agreement with our computed energy ( eV) for photoionizing an electron out of the  charge state and placing it in the conduction band. This observation is consistent with results on the optical absorption spectrum of Cr-doped GaN.^27^ An interesting correlation between the 1.5–2.0 eV absorption band responsible for green coloring of GaN and the sharp emission at 1.2 eV was found in GaN grown by hydride vapor phase epitaxy (HVPE).^28^ In addition, both of these optical signatures were found to correlate with the Cr concentration as measured by secondary ion mass spectrometry.

For Cr_Al_ in AlN we find a (+/0) level at 2.60 eV above the VBM. Additionally, Cr_Al_ can occur in negative charge states, with a (0/−) level at 3.83 eV above the VBM, and a (−/2−) level at 5.42 eV above the VBM [0.77 eV below the conduction-band minimum (CBM)]. These negative charge states, which do not occur in GaN, can be stabilized because of the larger band gap of AlN. These negatively charged Cr_Al_ states are stable in high-spin configurations, i.e., quintet *S* = 2 for 1– and sextet *S* = 5/2 for 2– charge states.

In our discussion of formation energies, we need to distinguish between different charge states of the centers. Each of these charge states corresponds to a particular oxidation state. For instance, when Cr substitutes on an Al or a Ga site, three electrons are needed to satisfy the bonding, leaving Cr in a 3+ oxidation state when the center is neutral. To avoid confusion with the charge-state notation, we use “Cr(III)” as the notation for the oxidation state. Cr(III) has a *d*^3^ occupation of the 3*d* states. We find that this leads to a spin state *S* = 3/2. The positive charge state corresponds to a Cr(IV) oxidation state with a *d*^2^ electron configuration, for which we find *S* = 1.

Turning to manganese, substitutional Mn can exist in 2+, 1, 0 and 1– charge states in both GaN and AlN (see Figure 1). In GaN the (+/0) charge-state transition level of Mn_Ga_occurs at 1.06 eV above the VBM, in very good agreement with the level at 1.11 eV that was experimentally determined from PL and PL excitation spectroscopy by Han et al.^29^ and predicted by Gerstmann et al.^30^ The computed (0/−) level of Mn_Ga_ occurs at 2.07 eV above the VBM, in reasonable agreement with an estimated value of 1.8 eV based on electron spin resonance and photoionization experiments by Graf et al.^31^

The neutral charge state of substitutional Mn in AlN and GaN, corresponding to Mn(III), has a *d*^4^ electronic configuration. We find that this leads to a high spin state of *S* = 2. For the negative charge state, corresponding to Mn(II) oxidation state, with a *d*^5^ electron configuration, we find *S* = 5/2. The quintet state (*S* = 2) of  is 1.25 eV lower in energy than the competing triplet (*S* = 1), in qualitative agreement with the result for cubic GaN reported in ref.^32^. For  the quintet stabilization energy is 1.39 eV.

In AlN, the (+/0) level of Mn_Al_ occurs at 1.33 eV above the VBM, and the (0/−) level at 2.40 eV above the VBM.

**Figure 2:**
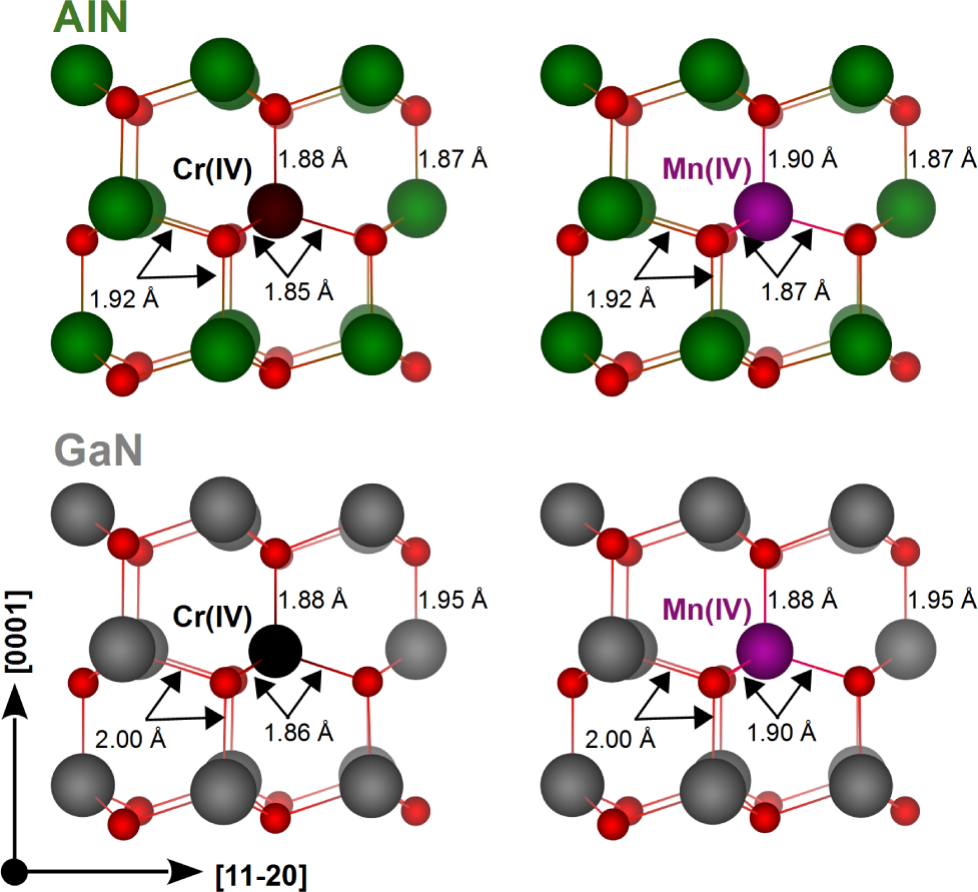
Ground state geometry of the investigated centers in the 1+ charge state.

Figure 2 illustrates the atomic structure of Cr(IV) and Mn(IV) (corresponding to the positive charge state) in AlN and GaN. The system preserves tetrahedral coordination and the relaxations of the surrounding nitrogen atoms are quite modest, which can be explained by almost identical ionic radii of Al(III) = 0.54 Å and Cr(IV) = 0.55 Å.^33^ In the case of Cr(IV) () in AlN, one Cr–N bond is 0.5% elongated and three Cr–N bonds are 1% shortened. Values for the nearest-neighbor bond lengths, also for the neutral charge state, are included in Table 1.

**Table 1:** Nearest-Neighbor Bond Lengths for the Cr and Mn Substitutional Impurities in Different Charge States *q^a^*

		Bond Length, Å	
Impurity	*q*	(TM–N)_∥_	(TM–N)_⊥_
Cr_Al_	1+	1.88	1.85
	0	1.96	1.95
Cr_Ga_	1+	1.88	1.86
	0	1.96	1.97
Mn_Al_	1+	1.90	1.87
	0	1.91	1.97
Mn_Ga_	1+	1.88	1.90
	0	1.90	1.99

a The bond lengths between the transition metal (TM) and the nearest neighbor N atoms are indicated by (TM–N)_∥_ for the bond along the *c* axis and (TM–N)_⊥_ for the other three bonds (which have equal length).

Different oxidation states of transition-metal centers can be stabilized in AlN or GaN, depending on the Fermi-level position. In GaN, which is easily *n*-type doped,  and  should be easy to form. In addition, experiments have reported unambiguous detection of both Cr(IV) and Mn(IV).^19,20,29^ From a practical perspective, we propose two strategies to stabilize the IV oxidation state: (1) doping with acceptors (e.g., Mg in GaN and Be in AlN) to move the Fermi level toward the valence band. This strategy has proven successful in stabilizing Mn(IV) in Mn–Mg-codoped GaN grown by metalorganic vapor phase epitaxy.^29^ (2) Performing the crystal growth under nitrogen-poor conditions. As demonstrated in a recent publication,^34^ Cr_Ga_^+^ is then stable for Fermi levels of 2.2 eV or less, thus favoring Cr(IV) over Cr(III).

### Ground-State Electronic Structure of Cr_X_ and Mn_X_ Defects in AlN and GaN

We discuss the electronic structure of Cr_X_ and Mn_X_ centers in AlN and GaN by using group theory and our calculated KS eigenvalue spectra. We focus on two specific *d* orbital occupations: *d*^2^ of ,  [Cr(IV) oxidation state] and *d*^3^ of , , ,  [Cr(III) and Mn(IV) oxidation states]. It should be noted that  also corresponds to a *d*^2^ configuration; however, the 2+ charge state is only stable for Fermi levels close to the VBM. Achieving such a Fermi level is unlikely for AlN, but GaN samples intentionally doped with Mg could have the Fermi level pinned in the desired range.^35^

**Figure 3:**
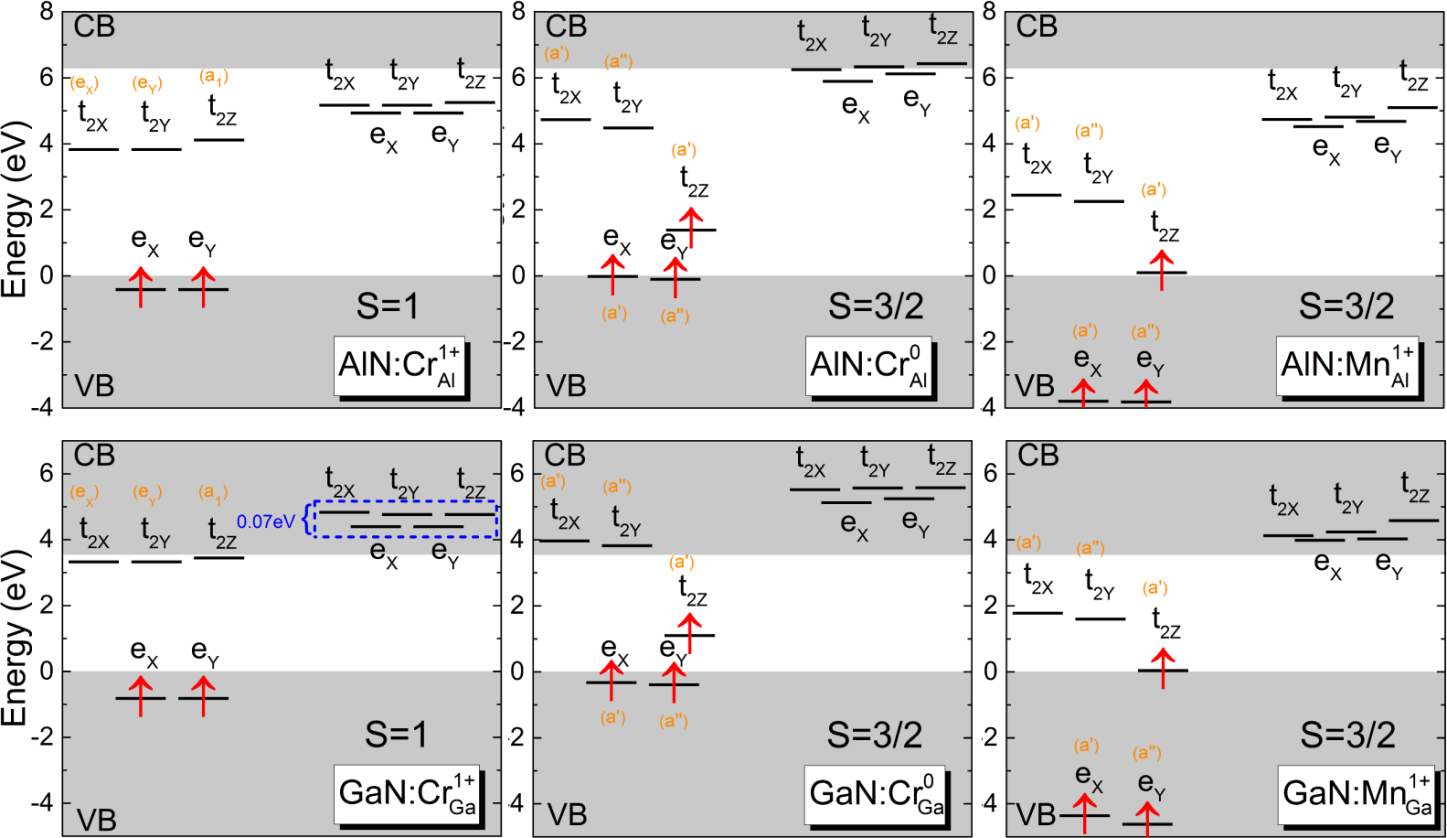
HSE KS eigenvalue spectra of the investigated centers in AlN and GaN. The gray shaded areas represent valence and conduction bands. Spin-up and spin-down channels are depicted and occupied states are represented by red arrows. The main symmetry labels associated with the *T*_d_ point group are displayed in black while the smaller orange labels in parentheses reflect the small symmetry breaking in the wurtzite structure (*C*_3v_ point group for *d*^2^ configuration) and the static Jahn–Teller effect (*C*_1_*_h_* point group for *d*^3^ configuration). The zero of energy is set at the VBM.

The KS eigenvalue diagrams of the impurity-related electronic states are shown in Figure 3. We first analyze the ground state of  and  in AlN and GaN as these defects are particularly attractive candidates for spin qubits. In a tetrahedral crystal field (*T*_d_), the 5-fold degenerate 3*d* orbitals split into lower-energy doubly degenerate *e* and higher-energy triply degenerate *t*_2_ states. Centers with the 3*d*^2^ (*e*^2^) electron configuration, i.e.,  and , have the highest point group symmetry and a triplet *S* = 1 spin state. The hexagonal crystal field of the wurtzite lattice causes a symmetry lowering from *T*_d_ to *C*_3*v*_ leading to a small splitting of the empty *t*_2_ states into doubly degenerate *e* and nondegenerate *a*_1_ states, split by 0.29 eV in AlN and 0.11 eV in GaN, consistent with previous work on .^36^Figure 3 shows that the occupied (spin-up) *e* states overlap with the valence band for both hosts. In AlN, all unoccupied states reside in the bandgap, whereas in GaN, the spin-majority-channel *t*_2_ states lie just below the CBM, and the spin-minority-channel *e* and *t*_2_ states overlap with the conduction band.

Adding one extra electron to the 3*d* manifold results in a 3*d*^3^ () electron configuration, which describes the neutral  and , and the positively charged  and . Our HSE calculations yield a high-spin *S* = 3/2 ground-state solution, satisfying Hund’s first rule (see Figure 3), consistent with the DFT investigation of transition-metal centers in cubic GaN by Schultz et al.^32^ The sizable magnetic moment on Cr and Mn induces a small antiferromagnetic polarization of about −0.04 μB on the nearest-neighbor N atoms. The  configuration is subject to a Jahn–Teller instability on top of the hexagonal crystal field, which further lowers the symmetry to *C*_1*h*_; the doubly degenerate *e* transforms to nondegenerate, symmetric *a*′ and asymmetric *a*″, and the triply degenerate *t*_2_ transforms to two symmetric *a*′ and one asymmetric *a*″ states.

### Excited States of Cr_X_ and Mn_X_ Defects in AlN and GaN

For the ground state, our HSE calculations are reliable, but the excited-state electronic structure of transition metals frequently requires going beyond the single Slater-determinant picture inherent in the generalized KS theory. As mentioned in Methods section, we apply the multideterminant cDFT (mcDFT for short) method, and corroborate our results with the CASCI method based on a cluster model. In Supporting Information, we describe how the eigenstates are explicitly written in terms of single-Slater-determinant states and provide expressions for the total energy. We specifically consider two excited states, namely the lowest spin-conserving and spin-flip excited states within the *d*^2^ and *d*^3^ manifolds. The results are summarized in Table 2. We note that the CASCI values in Table 2 are vertical excitation energies calculated at fixed ground-state geometry of the cluster.

**Table 2:** Vertical Transition Energies between the Ground State and the Two Lowest-Energy Excited States Description for Cr and Mn Substitutional Centers in AlN and GaN^a^

	ν, eV		ν, eV	
state	mcDFT	CASCI	mcDFT	CASCI
	AlN:		GaN:	
^3^*A*_2_	0.00	0.00	0.00	0.00
^1^*E*	1.25	1.26	1.24	1.27
^3^*T*_2_	1.83	1.92	1.77	1.98

	AlN:		GaN:	
^4^*T*_1_	0.00	0.00	0.00	0.00
^2^*E*	0.82	0.78	0.73	0.82
^4^*T*_2_	1.19	1.18	1.16	1.29

	AlN:		GaN:	
^4^*T*_1_	0.00	0.00	0.00	0.00
^2^*E*	0.75	0.83	0.70	0.86
^4^*T*_2_	1.01	1.09	0.98	1.11

a The energy of the ground state is used as the reference (set to zero). Values obtained with the mcDFT approach (based on single-Slater-determinant constrained HSE calculations) are compared with results from the CASCI approach.

A key conclusion is that the constrained-DFT results are in excellent agreement with the CASCI values (obtained with the *n*-electron valence state perturbation theory (NEVPT2) method), thereby lending confidence to both approaches.

Our results for *d*^2^ () yield an orbital-singlet, spin-triplet ground state of ^3^*A*_2_ symmetry, followed by an orbital-doublet, spin-singlet excited state ^1^*E*, and an orbital-triplet, spin-triplet excited state ^3^*T*_2_. We find the same ordering in AlN and in GaN. This order is consistent with the Tanabe–Sugano diagram for a *d*^2^ configuration in a tetrahedral crystal field,^13,26^ taking into account that the Tanabe–Sugano diagram for a *d*^*n*^ configuration in a tetrahedral crystal field is equivalent to that for a  configuration octahedral crystal field, and that  = 4/9. The energy difference between the ground and excited state of the same spin multiplicity is dominated by the crystal field splitting between *e* and *t*_2_ orbitals, whereas in the case of a spin-flip transition it is dominated by the change in Coulomb interaction.

For  we find that the ^1^*E* excited state lies 1.25 eV (mcDFT)/1.26 eV (CASCI) above the ^3^*A*_2_ ground state, in very good agreement with the experimental ZPL value of 1.201 eV.^20^ For , the ^1^*E* excited state lies 1.24 eV (mcDFT)/1.27 eV (CASCI) above the ^3^*A*_2_ ground state, again in very good agreement with the experimental ZPL value of 1.193 eV.^20^ As we will demonstrate in next section, the Franck–Condon shift of the ^1^*E* → ^3^*A*_2_ optical transition is negligibly small; therefore, the excitation and emission energies are nearly the same, and vertical transition energies are close to the ZPL.

For the lowest spin-conserving ^3^*T*_2_ → ^3^*A*_2_ transition we find an energy of 1.83 eV (mcDFT)/1.92 eV (CASCI) for , and 1.77 eV (mcDFT)/1.98 eV (CASCI) for .

In the case of 3*d*^3^, the electronic structure gets more complex as an additional electron occupies *t*_2_, forming a Jahn–Teller  unstable configuration in the ground state. Both the mcDFT and CASCI simulations yield the orbital-triplet, spin-quartet state of ^4^*T*_1_ symmetry as the ground state, followed by the orbital-doublet, spin-doublet excited state of ^2^*E* symmetry, and the orbital-triplet, spin-quartet excited state of ^4^*T*_2_ symmetry. The eigenstates of these multiplets are explicitly written in Supporting Information.

This calculated energy ordering is consistent with the Tanabe–Sugano diagram for *d*^3^ ion in *T*_d_ environment, for small  values. Again, we find the same ordering in AlN and GaN.

For the neutral Cr defect, we find spin-conserving transitions at 1.19 eV (mcDFT)/1.18 eV (CASCI) for  and 1.16 eV (mcDFT)/1.29 eV (CASCI) for . For  and , we find the first spin-conserving excited state to be at 1.01 eV (mcDFT)/1.09 eV (CASCI) in AlN and at 0.98 eV (mcDFT)/1.11 eV (CASCI) in GaN. For , our calculated relaxation energy upon excitation from Δ*S*CF is ∼0.13 eV; if we subtract this value, the predicted emission at 0.98 eV (CASCI) for the ^4^*T*_2_ → ^4^*T*_1_ transition matches the experimental value of 1.00 eV^29^ nearly perfectly.

These spin-conserving transitions are actually not the lowest-energy excitations. As can be seen in Table 2, we find a highly correlated excited-state doublet of ^2^*E* symmetry and  electron occupation at an energy in the range of 0.78–0.86 eV above the ground state for all the *d*^3^ cases. The lowest-energy transition is thus a ^2^*E* → ^4^*T*_1_ spin-flip transition; the excitation promotes the single *t*_2_ electron in the spin-majority channel to the empty *e* state in the spin-minority channel. Both the  and  are subject to Jahn–Teller effects, and we thus expect noticeable atomic distortions following the transition and pronounced Franck–Condon shifts, in contrast to the ^1^*E* → ^3^*A*_2_ optical transition in 3*d*^2^ case. This explains the lack of experimental detection of sharp single-photon emission originating from Cr(III) and Mn(IV) ions in AlN and GaN crystals.

### Emission Spectra of Cr_X_ and Mn_X_ Defects in AlN and GaN

**Figure 4:**
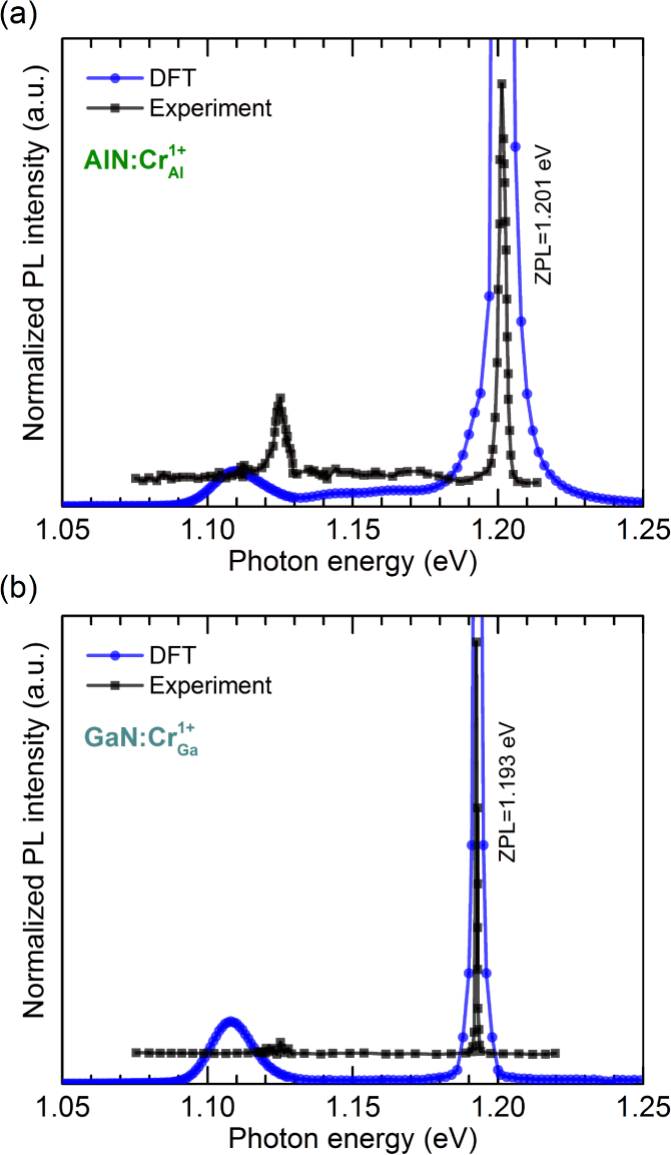
Calculated photoluminescence lineshapes associated with the ^1^*E* → ^3^*A*_2_ optical transition of (a)  in AlN and (b)  in GaN. The calculated spectra are shifted to match the experimental ZPL values.^20^

We now discuss the PL spectra associated with the lowest-energy transitions. The simulated PL lineshapes for the ^1^*E*^3^*A*_2_ optical transitions of  and  (with *d*^2^ configuration) are depicted in Figure 4. The calculated spectra are shifted to match the experimental ZPLs.^20^ Our simulations predict a sharp ZPL and a small phonon replica at approximately 75 meV below the ZPL. These features agree well with the experimental PL spectrum of ref.^20^, also shown in the figure, which was measured at ∼2 K on GaN grown on a sapphire substrate. A nearly identical PL spectrum for the Cr(IV) ^1^*E* → ^3^*A*_2_ transition was more recently measured by Koehl et al.^19^ at 30 K on a commercial bulk GaN substrate grown by HVPE, demonstrating that the substrate does not impact the spectral characteristics of the transition. We also note that ref.^28^ found (again in HVPE samples) that going from 15 to 294 K only slightly broadened the ZPL and shifted it from 1.193 to 1.187 eV, implying that Cr(IV) may be an efficient quantum emitter even at room temperature. We find that the phonon features originate from a quasilocal vibrational mode of *E* symmetry associated with the vibration of the nearest-neighbor N atoms. The calculated inverse participation ratio (IPR) values of these modes are in the range of 0.015–0.030 for both cases, at least 1 order of magnitude higher than the remaining bulk-like vibrations.

Both the simulated and the experimental spectra indicate a large Debye–Waller factor. Our simulations yield *w*_*ZPL*_ = 0.97 [*S*(0)=0.022] for  and *w*_*ZPL*_ = 0.96 [*S*(0) = 0.038] for . The reason for the high Debye–Waller factor is the negligible atomic relaxation of the excited state ^1^*E* as the transition flips an electron from the *e* spin-majority channel to the same *e* in the spin-minority channel. As a result, two electrons are distributed over two spin-up and spin-down orbitals, leading to nearly identical charge distributions in both ^3^*A*_2_ and ^1^*E*. This implies that the restoring forces between these two configurations are close to zero and the atomic structure stays the same. This feature, characteristic of a *d*^2^ system in tetrahedral coordination, is very desirable for high-efficiency single-photon emitters in solid-state systems.^37^

**Figure 5:**
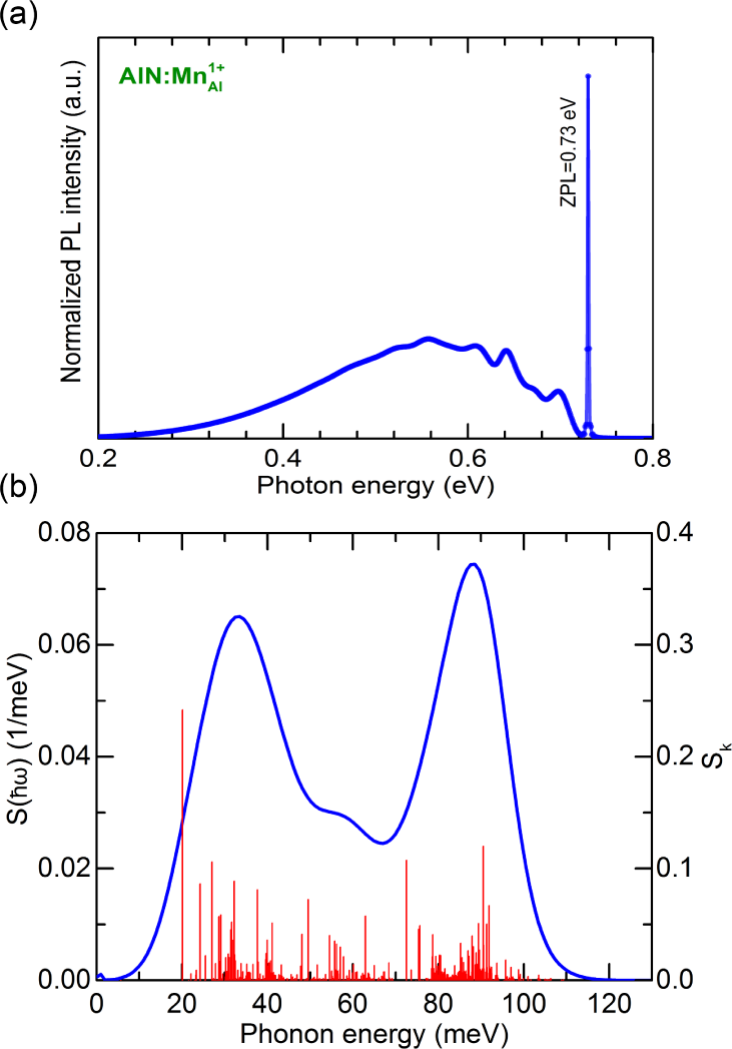
(a) Calculated photoluminescence line shape associated with the ^2^*E* → ^4^*T*_1_ optical transition of  in AlN. (b) Partial Huang–Rhys factor *S*_k_ in red and spectral function of electron–phonon coupling  in blue.

This case can be contrasted with the ^2^*E* → ^4^*T*_1_ optical transition (which occurs in the *d*^3^ systems), which shows a vastly different PL spectrum (see Figure 5). As an example, we computed the PL line shape associated with this transition for . In this case, a *t*_2_ electron in the spin-majority channel is promoted to an empty *e* state in the spin-minority channel. The change of orbital character during excitation significantly changes the charge distribution around the transition metal, resulting in a large structural relaxation. As a result the PL line shape has a broad phonon sideband [see Figure 5a]. By analyzing the spectral function of the electron–phonon coupling  and the partial HR factors *S*_k_, we can distinguish two major vibrational features that couple to the ^2^*E* → ^4^*T*_1_ transition. The first one is a low-energy quasilocal mode at 20 meV [*S*_k_ = 0.24] associated with the motion of the Mn atom. The second is a higher-energy bulk-like phonon mode at ∼90 meV.

The calculated total HR factor *S*(0) = 3.89 yields a low Debye–Waller factor *w*_*ZPL*_ = 0.02. While the NV center in diamond has a similar DW factor, the low DW factor combined with the spin-flip nature of the transition makes the center unattractive as a quantum emitter.

Our results highlight important aspects of utilizing transition-metal impurities for single-photon emission. Transition metals are often touted as having sharp emission lines with large Debye–Waller factors, but our results show that this is definitely not universally true: as exemplified by , some transitions within transition-metal impurities couple strongly to phonons and result in a small Debye–Waller factor.

This is detrimental not only because of reduced emission into the ZPL, but also because the large electron–phonon coupling potentially enables nonradiative recombination mechanisms that reduce the efficiency of the center.^37^ As shown by the case of , the large Debye–Waller factor is tied to the transition corresponding to a spin flip of a single orbital. While advantageous for reducing coupling to phonons, the need for a spin flip results in a slow radiative transition rate, demonstrating the trade-off inherent in the choice of optimal centers.

### Zero-Field Splitting of Cr_X_

Table 3 summarizes the calculated ZFS parameters for different charge states of Cr_X_ in AlN and GaN. For , the Cr(IV) cation (IV denotes the oxidation state) yields a spin-triplet state (*S* = 1) with a *d*^2^ (*e*^2^) electron configuration in the ^3^*A*_2_ ground state. Since the doubly degenerate *e* states are half-filled (occupied within the spin-majority channel, empty in the spin-minority channel) the SOC is weak due to the quenched orbital moment. As a result, the ZFS of  is dominated by the spin–spin contribution. Our HSE calculations indeed produced modest values for the calculated *D*, 4.5 GHz for  and 2.4 GHz . The value for  compares favorably with the measured value of 6.9 GHz for Cr(IV) in GaN.^19^

**Table 3:** Calculated ZFS (*D*, GHz) for Cr_X_ in AlN and GaN

	AlN		GaN	
				
*S*	1	3/2	1	3/2
*D* (HSE+SOC)	4.5	376	2.4	581
*D* (ORCA)	10.5	387	8.7	520
*D* (experiment)	–	–	6.9	–

For , we find much larger *D* values. The 3*d*^3^ electron configuration corresponds to  orbital occupation, and the partial filling of the *t*_2_ orbitals increases SOC (see ref.^38^ for a discussion of the band-filling effect on magnetic anisotropy). Our calculated values are 376 GHz for  and 581 GHz for .

As a check, we have also calculated the ZFS with the CASCI approach, using the EPR/NMR module of the ORCA package. As can be seen in Table 3, we obtain satisfactory agreement between the two methods.

### Realization of a Spin Qubit Based on  or 

We now demonstrate that the  and  centers with *d*^2^ orbital occupation can serve as a platform for the realization of a solid state spin qubit. We have already shown that the centers with *d*^3^ occupation give rise to strong electron–phonon coupling that limits emission into the ZPL. In addition, the *d*^3^ electron configuration induces very large ZFS dominated by SOC, which is unsuitable for pulsed microwave resonance manipulation (see the previous section). We conclude that neither  or  in AlN, nor  or  in GaN, are good candidates for a single photon source or quantum bit.

We therefore focus on the  and  centers, which have a desirable orbital-singlet, spin-triplet ^3^*A*_2_ ground state that minimizes SOC, and a wide charge-stability window in AlN and GaN. As seen in Figure 1, stabilizing the 1+ charge state requires moving the Fermi level below 2.60 eV in AlN or below 2.21 eV in GaN, which can be accomplished by some form of acceptor doping. Most of the emission from the first excited state ^1^*E* is contained within the narrow ZPL optical transition with no competing intermediate states.

**Figure 6:**
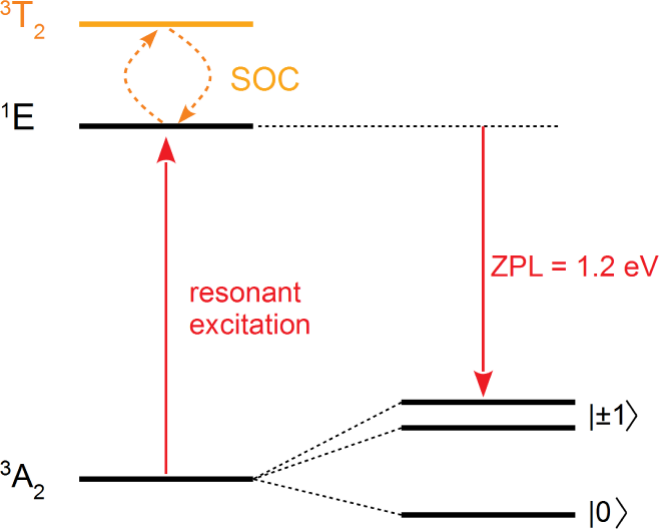
Optical spin polarization cycle for  in AlN or  in GaN.

We find ZFS values of a few GHz, which is suitable for pulsed microwave resonance manipulation of the electron spin between  and  in the ground state. The optical spin polarization cycle is illustrated in Figure 6. Considering selection rules, the transition rate for the ^1^^3^ transition will be higher than for ^1^^3^, and this difference in rates can be used to optically initialize the qubit. By applying continuous excitation at ∼1.2–1.3 eV, spin polarization of the ground state can be achieved. This value of the excitation energy will avoid exciting the spin-conserving^3^*T*_2_ state, as well as photoionization of the center.

## Conclusions

In summary, we investigated the ground and excited state properties of chromium and manganese substitutional centers in wurtzite AlN and GaN to explore their potential in nanophotonics and quantum information processing applications. Based on the formation energies, we unravel the stability windows and the charge-state transition levels of each center, achieving excellent agreement with the available experimental data for Cr- and Mn-doped AlN and GaN. For excited states, we compared the multideterminant constrained DFT (mcDFT) approach with the complete active space configuration interaction (CASCI) method, finding good agreement. We note that the DFT-based approach has the ability to calculated relaxation energies, offering more detailed insight in differences between zero-phonon lines and vertical transition energies for emission and absorption.

Regarding the magneto–optical properties, we focused our analysis to those defects exhibiting 3*d*^2^ and 3*d*^3^ electron configuration, i.e., , , and . The simulated PL spectra confirm the atomistic origin of sharp infrared transitions near 1.2 eV observed experimentally for Cr-doped AlN and GaN and their relation to the  center. Through the combination of the electronic structure analysis of the ground state and the lowest excited states, PL lineshapes, and ZFS parameters, we prove that  can serve as a single-photon source and spin qubit. The other transition-metal-related centers considered here are less promising candidates, which we attribute to the *d*^3^ electron configuration that (in a tetrahedral bonding environment) leads to stronger electron–phonon coupling and larger ZFS.

## Methods

### Electronic Structure and Ground-State Atomic Configuration

DFT calculations within the generalized KS scheme were carried out using the Vienna Ab Initio Simulation Package (VASP).^39,40^ Valence electrons were separated from the core by application of projector augmented wave (PAW) potentials.^41^ Gallium *d* states were were treated as core states. A plane-wave cutoff energy of 450 eV was applied. To correctly characterize defect-related states, the bandgap of the material needs to be accurately produced in the calculations. Traditional functionals such as the local density approximation (LDA) or the generalized gradient approximation (GGA) underestimate the bandgap; here we employed the screened range-separated hybrid functional of Heyd, Scuseria, and Ernzerhof (HSE).^42,43^ We set the fraction of exact exchange to α_AlN_ = 0.32 and α_GaN_ = 0.30 while keeping the default screening parameter ω = 0.2 1/Å. With these choices we obtained a bandgap of 6.19 eV for AlN and 3.55 eV for GaN, reproducing the experimental values.^44^ The calculated lattice constants of *a*_AlN_ = 3.09 Å, *c*_AlN_ = 4.93 Å and *a*_GaN_ = 3.19 Å, *c*_GaN_ = 5.19 Å also agree well with reported experimental values.^45^ The bond lengths in the bulk are 1.885 Å for (Al–N)_∥_ and 1.876 Å for (Al–N)_⊥_ in AlN, and 1.959 Å for (Ga–N)_∥_ and 1.950 Å for (Ga–N)_⊥_ in GaN, where we denote with “” the bond along the *c* axis, and with “” the other three bonds.

Defect calculations were performed in a supercell geometry. Convergence as a function of supercell size was tested by comparing results in 96-atom supercells with 300-atom supercells. Gratifying agreement was found, for instance, vertical transitions energies agreed to better than 0.02 eV. All results reported in the paper are based on the large 300-atom (5 × 5 × 3) wurtzite supercell. This enables accurate sampling of the first Brillouin zone using the Γ point, for which the KS wave functions possess the full symmetry of the system. Defects in the supercell were relaxed at constant volume until the Hellmann–Feynman forces acting on each atom dropped below 0.01 eV/Å. Spin polarization was included. We performed atomic relaxations for seven possible charge states of each defect (3+, 2+, 1+, 0, 1–, 2–, 3−) to capture all stable charge states and charge-state transition levels. In diagrams such as Figure 1, only the lowest-energy charge state at each Fermi-level position is shown.

### Formation Energies and Transition Levels

We computed the formation energy () of a defect using the standard formula:^46^
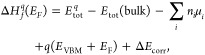
1where *q* denotes the charge state of the defect,  is the total energy of the supercell containing the defect,  is the total energy of the supercell containing pristine bulk, *n*_i_ indicates the number of atoms of type *i* (either host or impurity atoms) that were added to () or removed from () the supercell to create the defect, μ_*i*_ is the chemical potential of the corresponding atoms (*i* = Al, Ga, N, O, Cr, Mn), and the Fermi level *E*_F_ is referenced to the valence-band maximum (VBM) of pristine AlN or GaN. The chemical potential μ_N_ represents the energy of the reservoir with which nitrogen atoms are exchanged, reflecting the experimental conditions. It may vary between N-rich (Al- or Ga-poor) and N-poor (Al- or Ga-rich) extremes, with bounds set by the computed formation enthalpy of AlN: μ_Al_+μ_N_ =  = −3.19 eV and GaN: μ_Ga_+μ_N_ =  = −1.11 eV. For Cr, we use the energy of the body-centered cubic phase of bulk Cr, with antiferromagnetic ordering. We also calculated CrN, but this phase does not limit the Cr chemical potential under the cation-rich conditions for which we will present results. The last term  denotes the finite-size correction according to the Freysoldt correction scheme.^47,48^

The thermodynamic charge-state transition level  can be defined as the Fermi level position below which the defect is stable in the charge state *q*_1_ and above which it is stable in charge state *q*_2_. It is calculated as
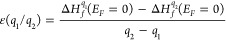
2

### Zero-Field Splitting Parameters

ZFS parameters for different charge states of the defect were computed from first principles. Both spin–spin dipolar interactions and SOC may contribute to ZFS.^49^ The spin–spin dipolar contribution is calculated as described in refs.^50−54^, while the ZFS induced by SOC is calculated by mapping the fully relativistic total energies (with SOC included) of different spin orientations to the spin Hamiltonian.^51−53^ Details are given in Supporting Information. The SOC contribution to the ZFS is analogous to calculating the single-ion anisotropy for the spin state of the defect.

The spin Hamiltonian with second-order terms is defined as
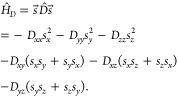
3The parameters *D*_*αβ*_ can be computed by the four-state mapping method:^52,53^
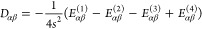
4where *E*_*αβ*_ are fully relativistic total energies for different spin orientations (see the Supporting Information of ref.^53^ for more details). All energies are obtained from HSE calculations. The diagonalization of the  matrix yields a traceless tensor *D*^′^:
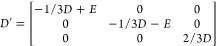


Within *C*_3*v*_ symmetry, which applies to all the studied defects here, *E* = 0 and we only report the *D* value in Results and Discussion. The *z* direction is defined to be along the high-symmetry axis of the defect, which coincides with the *c* axis of the wurtzite structure.

### Excited-State Electronic Structure and Optical Signatures

To calculate the excited-state energies of the defects, we first applied the constrained-DFT Δ-Self-Consistent Field (ΔSCF) method implemented in the VASP code. This approach allows one to determine the excitation energy, ZPL, and the relaxation energy upon optical excitation. Given that the generalized KS theory is based on single Slater determinants, care needs to be taken to account for the multideterminant nature of the investigated excited states. This issue can be overcome by combining energies from several single-determinant calculations, as proposed by Ziegler^55^ and implemented by von Barth^56^ within DFT. The single-Slater-determinant calculations are carried out with constrained DFT (cDFT), and we refer to this approach as mcDFT. This approach has recently been applied to several defects, resulting in good agreement with experiment.^8,13,57,58^ To verify the accuracy of our computed results within the mcDFT treatment, we also applied the CASCI method using cluster geometries. The studied clusters are based on the crystal structure geometries described above. The cluster shape and size for multiconfigurational calculations were chosen so that they were small enough to simulate them using memory-consuming techniques, but sufficiently large to describe the properties of the extended system and the crystal-field splitting. 48-Atom clusters (24 cations and 24 nitrogen atoms) were cut from the HSE-optimized geometries, and the positions of all host and impurity atoms were kept fixed in the CASCI calculations. The surfaces of the clusters need to be passivated to eliminate surface states; this was accomplished by saturating all surface dangling bonds with hydrogen atoms. The positions of the hydrogen atoms (which are not critical to the results, as long as they passivate the surface of the cluster) were optimized using the Perdew–Burke–Ernzerhof (PBE)^59,60^ functional. Additional information about the cluster model used in the CASCI calculations is given in Supporting Information.

Initial orbitals were constructed using the so-called fragment-derived guess. The cluster was fragmented into a cluster without a defect atom (i.e., a cluster representing bulk with a cation vacancy) and a defect metal atom. Molecular orbitals were obtained for each fragment: cluster orbitals were obtained from DFT and metal orbitals from CASCI. Then the resulting orbitals were merged. This approach allows for the use of orbitals based on pure metal in the calculation.

The multireference calculations for the cluster systems were performed using the ORCA package^61,62^ with a cc-pVDZ basis set. We applied the resolution-of-the-identity chain-of-spheres exchange (RIJCOSX)^63^ algorithm that treats the Coulomb term via repulsion integrals and the exchange term via seminumerical integration. Corrected energetic states for clusters were found using the strongly contracted NEVPT2 method.^64^

### Vibronic Coupling and Photoluminescence Spectrum

We applied density functional perturbation theory (DFPT) to compute the phonon spectrum of each defect. We used the PBE functional,^59,60^ which is computationally more efficient and has been shown to be adequate for obtaining accurate vibrational properties.^65^ To calculate the vibrational modes with high precision, we applied a strict 10^–4^ eV/Å force convergence criterion.

The normal modes can be analyzed using the IPR approach^66^ defined as
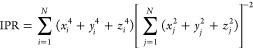
5where *N* denotes the number of atoms in the system, and  is the normalized 3*N*-dimensional vector of the atomic displacements of the corresponding vibrational eigenmode.

We calculated vibrational overlaps and investigated the photoluminescence (PL) spectrum within the Huang–Rhys (HR) theory.^67^ The computational implementation of HR theory was introduced by Alkauskas et al. and successfully demonstrated for defects in diamond.^68,69^ Details about the computational implementation of HR theory are given in Supporting Information.

## Supporting Information Available

First-principles evaluation of the *D* tensor; additional information about the cluster model used in the CASCI calculations; photoluminescence spectrum within the Huang–Rhys theory; mcDFT approach: multielectron eigenstates (PDF)
