# Supplementary material for: Transition-Metal-Related Quantum Emitters in Wurtzite AlN and GaN
Source: ACS Nano. 2024 Oct 12;18(42):28724–34. doi: 10.1021/acsnano.4c07184 (PMC11503773; doi:10.1021/acsnano.4c07184)
Supplement: Supplementary file 1 — nn4c07184_si_001.pdf [file nn4c07184_si_001.pdf]

Supporting Information for: Transition-metal-related quantum emitters in wurtzite AlN and GaN

Kamil Czelej,[*,†] M. Rey Lambert,[¶] Mark E. Turiansky,[§] Aleksei Koshevarnikov,[‖] Sai Mu,[⊥] and Chris G. Van de Walle[*,§]

†*Faculty of Chemical and Process Engineering, Warsaw University of Technology, Ludwika Warynskiego 1, 00-645 Warsaw, Poland*

‡*Institute of Theoretical Physics, Faculty of Physics, University of Warsaw, Pasteura 5, 02-093 Warsaw, Poland*

¶*Department of Physics, University of California, Santa Barbara, California 93106-9530, USA*

§*Materials Department, University of California, Santa Barbara, California 93106-5050, USA*

‖*Institute of Organic Chemistry, Polish Academy of Sciences, Kasprzaka 44/52, 01-224 Warsaw, Poland*

⊥*SmartState Center for Experimental Nanoscale Physics, Department of Physics and Astronomy, University of South Carolina, Columbia, South Carolina 29208, USA*

E-mail: kamil.czelej@pw.edu.pl; vandewalle@mrl.ucsb.edu

First-principles evaluation of the D tensor

The spin Hamiltonian in Equation (3) contains the components of the D tensor. The values can be computed by mapping the relativistic DFT energies of various spin orientations to this spin Hamiltonian, using the four-states mapping method. The four states corresponds to the spin moment on the Cr center being constrained to different directions. Each state is converged self-consistently with SOC included. The definition of each D component is shown below and the spin direction for each energy is specified.

$$D_{xy} = \frac{(E_{xy}^1 + E_{xy}^4) - (E_{xy}^2 + E_{xy}^3)}{4s^2} \tag{S1}$$

State	Spin directions
E_{xy}^1	$(\sqrt{2}/2, \sqrt{2}/2, 0)$
E_{xy}^2	$(\sqrt{2}/2, -\sqrt{2}/2, 0)$
E_{xy}^3	$(-\sqrt{2}/2, \sqrt{2}/2, 0)$
E_{xy}^4	$(-\sqrt{2}/2, -\sqrt{2}/2, 0)$

$$D_{xz} = \frac{(E_{xz}^1 + E_{xz}^4) - (E_{xz}^2 + E_{xz}^3)}{4s^2} \tag{S2}$$

State	Spin directions
E_{xz}^1	$(\sqrt{2}/2, 0, \sqrt{2}/2)$
E_{xz}^2	$(\sqrt{2}/2, 0, -\sqrt{2}/2)$
E_{xz}^3	$(-\sqrt{2}/2, 0, \sqrt{2}/2)$
E_{xz}^4	$(-\sqrt{2}/2, 0, -\sqrt{2}/2)$

$$D_{yz} = \frac{(E_{yz}^1 + E_{yz}^4) - (E_{yz}^2 + E_{yz}^3)}{4s^2} \tag{S3}$$

State	Spin directions
E_{yz}^1	$(0, \sqrt{2}/2, \sqrt{2}/2)$

E^2_{yz} $(0, \sqrt{2}/2, -\sqrt{2}/2)$

E^3_{yz} $(0, -\sqrt{2}/2, \sqrt{2}/2)$

E^4_{yz} $(0, -\sqrt{2}/2, -\sqrt{2}/2)$

$$D_{zz} - D_{xx} = \frac{(E^1_{(zz-xx)} + E^4_{(zz-xx)}) - (E^2_{(zz-xx)} + E^3_{(zz-xx)})}{4s^2} \tag{S4}$$

State Spin directions

$E^1_{(zz-xx)}$ $(0, 0, 1)$

$E^2_{(zz-xx)}$ $(1, 0, 0)$

$E^3_{(zz-xx)}$ $(-1, 0, 0)$

$E^4_{(zz-xx)}$ $(0, 0, -1)$

$$D_{yy} - D_{xx} = \frac{(E^1_{(yy-xx)} + E^4_{(yy-xx)}) - (E^2_{(yy-xx)} + E^3_{(yy-xx)})}{4s^2} \tag{S5}$$

State Spin directions

$E^1_{(yy-xx)}$ $(0, 1, 0)$

$E^2_{(yy-xx)}$ $(1, 0, 0)$

$E^3_{(yy-xx)}$ $(-1, 0, 0)$

$E^4_{(yy-xx)}$ $(0, -1, 0)$

Additional information about the cluster model used in the CASCI calculations

The cluster used in the CASCI calculations consists of 94 atoms: 23 Ga (Al), 1 transition metal (Cr, Mn), 24 N, and 46 H. The cluster is cut from the HSE-calculated supercells (with relaxed atomic positions) and is bounded by four $(\bar{1}\bar{1}20)$ and two (0001) crystallographic planes of the wurtzite structure. As shown in Fig. S1, surface (edge) atoms are passivated by one or two hydrogen atoms, ensuring 4-fold coordination of each Ga (Al) and N atom. The

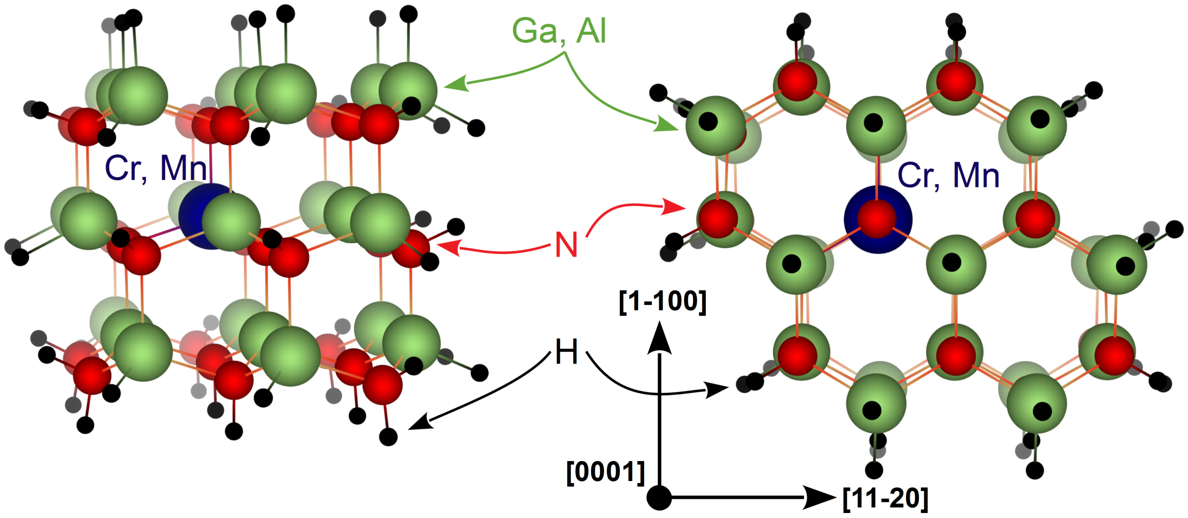

Figure S1: Atomic structure of cluster model used in CASCI calculations

selected size of the cluster results in odd and even number of electrons for defects in 1+ and 0 charge states, respectively, enabling correct prediction of spin states S=1 for 3d^2 and S=3/2 for the d^3 electron configurations in the ground state. The electronic structure of our cluster in the ground state resembles the electronic structure from the supercell HSE calculations, including the same order of VBM (HOMO), d states, and CBM (LUMO), enabling the active space for CASCI to be unambiguously selected. We define the active space as all five 3d orbitals and 2 electrons in case of $\mathrm{Cr}^{1+}_{\mathrm{Al,Ga}}$ or 3 electrons in case of $\mathrm{Cr}^{0}_{\mathrm{Al,Ga}}$ and $\mathrm{Mn}^{1+}_{\mathrm{Al,Ga}}$.

Photoluminescence spectrum within the Huang–Rhys theory

We calculate the normalized luminescence intensity, which is defined as follows

$$L(\hbar\omega) = C\omega^3 A(\hbar\omega), \tag{S6}$$

with the optical spectral function $A(\hbar\omega)$ defined as:

$$A(\hbar\omega) = \sum_m |\langle\chi_{gm}|\chi_{e0}\rangle|^2 \, \delta\left(E_{ZPL} - E_{gm} - \hbar\omega\right). \tag{S7}$$

The χ_{e0} and χ_{gm} are vibrational eigenstates of the excited and the ground state, and E_{gm} is the energy of the χ_{gm} state, which is the sum of individual energies over all vibrational modes k: $E_{gm} = \sum_k n_k \hbar\omega_k$. The ω^3 prefactor arises from the density of states of photons that cause the spontaneous emission ($\sim \omega^2$) and the perturbing electric field of those photons ($|\vec{\varepsilon}|^2 \sim \omega$), and C is the normalization constant

$$C^{-1} = \int A(\hbar\omega)\omega^3 d(\hbar\omega). \tag{S8}$$

The most demanding part of Eq. (S7) involves a series of multidimensional overlap integrals $\langle\chi_{gm}|\chi_{e0}\rangle$, that in practice, can be evaluated only for molecules, small clusters, or model defect systems. Vibrational modes contributing to $A(\hbar\omega)$ arise from the bulk lattice vibrations and from local or quasi-local modes induced by defects. In addition, the normal modes in the excited state can, in principle, differ from those in the ground state.[1] To simplify the evaluation of the spectral function $A(\hbar\omega)$ [Eq. (S7)], we assume that the normal modes that contribute to the luminescence line shape are the same in both the excited and ground states. The validity of this approximation was confirmed by the remarkable agreement between numerical and experimental results for the NV center in diamond,[1] even though the condition is not satisfied.

The optical spectral function defined in Eq. (S7) can then be reformulated using a generating function approach proposed by Lax[2] and by Kubo and Toyozawa.[3] A crucial quantity that should be determined is the spectral function of the electron–phonon coupling,[4] which is expressed as

$$S(\hbar\omega) = \sum_k S_k \delta\left(\hbar\omega - \hbar\omega_k\right), \tag{S9}$$

where the sum goes over all phonon modes k with frequencies ω_k. S_k is defined as the partial Huang-Rhys factor, given by

$$S_k = \frac{\omega_k q_k^2}{2\hbar}\,, \tag{S10}$$

where q_k is defined as

$$q_k = \sum_{\alpha,i} \sqrt{m_\alpha} \left(R^{(e)}_{\alpha,i} - R^{(g)}_{\alpha,i} \right) \Delta r_{k,\alpha,i}\,. \tag{S11}$$

m_α denotes the mass of atom α, $R^{(e)}_{\alpha,i}$ and $R^{(g)}_{\alpha,i}$ are equilibrium coordinates of an atom α along the direction i in the excited and the ground state, and $\Delta r_{k,\alpha,i}$ is the normalized displacement vector of an atom α along the direction i in the phonon mode k. Upon deriving the function $S(\hbar\omega)$, the optical spectral function can be expressed using the Fourier transform of the generating function $G(t)$:

$$A(E_{ZPL} - \hbar\omega) = \frac{1}{2\pi} \int_{-\infty}^{\infty} G(t) e^{i\omega t - \gamma|t|} dt, \tag{S12}$$

where γ represents the width of the ZPL. The generating function is defined as

$$G(t) = e^{S(t) - S(0)}, \tag{S13}$$

where $S(t)$ is the Fourier transform of the partial HR factor

$$S(t) = \int_0^{\infty} S(\hbar\omega) e^{-i\omega t} d(\hbar\omega). \tag{S14}$$

$S(0)$ denotes the total HR factor for a given optical transition and is defined as the sum of the partial HR factors over all phonon modes k:

$$S(0) = \int_0^{\infty} S(\hbar\omega) d(\hbar\omega) = \sum_k S_k. \tag{S15}$$

The total HR factor serves as a crucial parameter for characterizing the vibrational structure of the PL band. It is frequently employed for determining the weight of the ZPL[2,5,6]

$$w_{ZPL} = e^{-S(0)}. \tag{S16}$$

The quantity w_{ZPL} is called the Debye-Waller (DW) factor and describes the fraction of photons emitted into the ZPL.

mcDFT approach: Multielectron eigenstates

In general, multielectron wavefunctions can be expressed as a linear combination of two or more single Slater determinants.[7,8] In some cases, the total energy of such multideterminant states can be approximated by DFT. This approach involves computing the total energy for each individual Slater determinant by constrained DFT, where the occupation of target KS states is explicitly set and the structure is allowed to relax. To obtain reasonable approximations, one has to select proper KS wavefunctions (they should not be symmetry broken), and spin contamination should be avoided.[9]

The wavefunctions for each single Slater determinant are optimized, as in the original von Barth approach[7] within constrained DFT. Steps are taken to ensure the original symmetry is maintained. We start from pre-converged wavefunctions that have the correct symmetry. Subspace diagonalization within the electronic optimization is turned off (the "LDIAG = False" tag in VASP) to ensure that the orbital ordering and character is preserved. Finally, we inspect the final orbitals to ensure that they have maintained the correct character. To obtain the geometry of the excited state we utilize the forces of a single Slater determinant.

Here we explicitly write down the expressions for the ground state and two lowest excited states of d^2 ($^3A_2, ^1E, ^3T_2$) and d^3 ($^4T_1, ^2E, ^4T_2$) systems in a tetrahedral crystal field. The ν and μ are the components of e states, whereas ζ, ξ, η represent the components of t_2 states.

$3d^2$ electron configuration in a T_d crystal field

$$|^3A_2, m_s{=}\ 1\rangle = |\nu^{\uparrow}\mu^{\uparrow}\rangle \tag{S17}$$

$$|^3A_2, m_s{=}\ 0\rangle = \frac{1}{\sqrt{2}}(|\nu^{\uparrow}\mu^{\downarrow}\rangle + |\nu^{\downarrow}\mu^{\uparrow}\rangle) \tag{S18}$$

$$|^1E, m_s{=}\ 0\rangle = \frac{1}{\sqrt{2}}(|\nu^{\uparrow}\mu^{\downarrow}\rangle - |\nu^{\downarrow}\mu^{\uparrow}\rangle) \tag{S19}$$

$$|^3T_2, \zeta, m_s{=}\ 1\rangle = |\nu^{\uparrow}\zeta^{\uparrow}\rangle \tag{S20}$$

$$|^3T_2, \xi, m_s{=}\ 1\rangle = \frac{1}{2}|\nu^{\uparrow}\xi^{\uparrow}\rangle - \frac{\sqrt{3}}{2}|\mu^{\uparrow}\xi^{\uparrow}\rangle \tag{S21}$$

$$|^3T_2, \eta, m_s{=}\ 1\rangle = \frac{1}{2}|\nu^{\uparrow}\eta^{\uparrow}\rangle + \frac{\sqrt{3}}{2}|\mu^{\uparrow}\eta^{\uparrow}\rangle \tag{S22}$$

The approximate energy of the first singlet excited state ^1E can be obtained by linear combination of two single Slater determinants

$$E[^1E] = 2E[\nu^{\uparrow}\mu^{\downarrow}] - E[\nu^{\uparrow}\mu^{\uparrow}] \tag{S23}$$

where

$$E[\nu^{\uparrow}\mu^{\downarrow}] = \frac{1}{2}(E[^1E] + E[^3A_2]) \tag{S24}$$

and

$$E[\nu^{\uparrow}\mu^{\uparrow}] = E[^3A_2] \tag{S25}$$

$3d^3$ electron configuration in a T_d crystal field

$$|^4T_1, \xi, m_s{=}\ 3/2\rangle = |\nu^{\uparrow}\mu^{\uparrow}\xi^{\uparrow}\rangle \tag{S26}$$

$$|^4T_1, \eta, m_s{=}\ 3/2\rangle = |\nu^{\uparrow}\mu^{\uparrow}\eta^{\uparrow}\rangle \tag{S27}$$

$$|^4T_1, \zeta, m_s{=}\ 3/2\rangle = |\nu^{\uparrow}\mu^{\uparrow}\zeta^{\uparrow}\rangle \tag{S28}$$

$$|^4T_1, m_s\!=1/2\rangle\!= \frac{1}{\sqrt{3}}(|\nu^\uparrow\mu^\uparrow\xi^\downarrow\rangle + |\nu^\uparrow\mu^\downarrow\xi^\uparrow\rangle + |\nu^\downarrow\mu^\uparrow\xi^\uparrow\rangle) \tag{S29}$$

$$|^2E, m_s\!=1/2\rangle\!= |\nu^\uparrow\mu^\uparrow\nu^\downarrow\rangle \tag{S30}$$

$$|^4T_2, \zeta, m_s\!=3/2\rangle = |\nu^\uparrow\xi^\uparrow\eta^\uparrow\rangle \tag{S31}$$

$$|^4T_2, \xi, m_s\!=3/2\rangle\!= \frac{\sqrt{3}}{2}|\nu^\uparrow\zeta^\uparrow\eta^\uparrow\rangle + \frac{1}{2}|\mu^\uparrow\zeta^\uparrow\eta^\uparrow\rangle \tag{S32}$$

$$|^4T_2, \eta, m_s\!=3/2\rangle\!= \frac{\sqrt{3}}{2}|\nu^\uparrow\xi^\uparrow\zeta^\uparrow\rangle - \frac{1}{2}|\mu^\uparrow\xi^\uparrow\zeta^\uparrow\rangle \tag{S33}$$

In this case, one can calculate the energy of the first doublet excited state ^2E by calculating the total energy of a single constrained Slater determinant

$$E[^2E] = E[\nu^\uparrow\mu^\uparrow\nu^\downarrow] \tag{S34}$$

References

(1) Alkauskas, A.; Buckley, B. B.; Awschalom, D. D.; Van de Walle, C. G. First-Principles Theory of the Luminescence Lineshape for the Triplet Transition in Diamond NV Centres. *New Journal of Physics* **2014**, *16*, 073026.

(2) Lax, M. The Franck-Condon Principle and Its Application to Crystals. *J. Chem. Phys.* **2004**, *20*, 1752–1760.

(3) Kubo, R.; Toyozawa, Y. Application of the Method of Generating Function to Radiative and Non-Radiative Transitions of a Trapped Electron in a Crystal. *Prog. Theor. Phys.* **1955**, *13*, 160–182.

(4) Miyakawa, T.; Dexter, D. L. Phonon Sidebands, Multiphonon Relaxation of Excited States, and Phonon-Assisted Energy Transfer between Ions in Solids. *Phys. Rev. B* **1970**, *1*, 2961–2969.

(5) Markham, J. J. Interaction of Normal Modes with Electron Traps. *Rev. Mod. Phys.* **1959**, *31*, 956–989.

(6) Davies, G. The Jahn-Teller Effect and Vibronic Coupling at Deep Levels in Diamond. *Rep. Prog. Phys.* **1981**, *44*, 787.

(7) von Barth, U. Local-Density Theory of Multiplet Structure. *Phys. Rev. A* **1979**, *20*, 1693–1703.

(8) Shang, L.; Chen, Q.; Jing, W.; Ma, C.-G.; Duan, C.-K.; Du, J. First-Principles Study of Transition Metal Dopants As Spin Qubits. *Phys. Rev. Mater.* **2022**, *6*, 086201.

(9) Czelej, K.; Zemła, M. R.; Śpiewak, P.; Kurzydłowski, K. J. Quantum Behavior of Hydrogen-Vacancy Complexes in Diamond. *Phys. Rev. B* **2018**, *98*, 235111.
